# Dietary Advanced Glycation End Products and Aging

**DOI:** 10.3390/nu2121247

**Published:** 2010-12-13

**Authors:** Claudia Luevano-Contreras, Karen Chapman-Novakofski

**Affiliations:** Division of Nutritional Sciences, University of Illinois at Urbana-Champaign, Urbana, IL 61801, USA; Email: kmc@illinois.edu

**Keywords:** advanced glycation end products, aging, Maillard reaction

## Abstract

Advanced glycation end products (AGEs) are a heterogeneous, complex group of compounds that are formed when reducing sugar reacts in a non-enzymatic way with amino acids in proteins and other macromolecules. This occurs both exogenously (in food) and endogenously (in humans) with greater concentrations found in older adults. While higher AGEs occur in both healthy older adults and those with chronic diseases, research is progressing to both quantify AGEs in food and in people, and to identify mechanisms that would explain why some human tissues are damaged, and others are not. In the last twenty years, there has been increased evidence that AGEs could be implicated in the development of chronic degenerative diseases of aging, such as cardiovascular disease, Alzheimer’s disease and with complications of diabetes mellitus. Results of several studies in animal models and humans show that the restriction of dietary AGEs has positive effects on wound healing, insulin resistance and cardiovascular diseases. Recently, the effect of restriction in AGEs intake has been reported to increase the lifespan in animal models. This paper will summarize the work that has been published for both food AGEs and *in vivo* AGEs and their relation with aging, as well as provide suggestions for future research.

## 1. Introduction

Advanced glycation end products (AGEs) are a heterogeneous, complex group of compounds that are formed mainly via the Maillard reaction. The Maillard reaction occurs when reducing sugar reacts in a non-enzymatic way with amino acids in proteins, lipids or DNA. This reaction has been studied for years in the food industry because its products add a desirable color and taste to foods. However, the study of the products of this reaction *in vivo* have received increasing attention in recent years due to association of AGEs with certain chronic diseases, such as diabetes mellitus, cardiovascular diseases, and Alzheimer’s disease, as well as during the aging process.

## 2. Formation of AGEs

**Figure 1:**
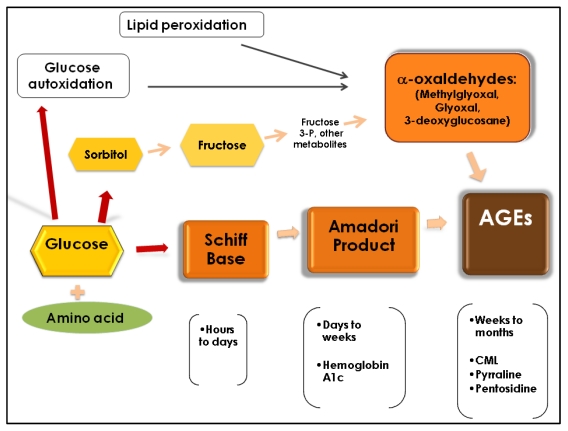
Formation of AGEs.

The formation of AGEs through the Maillard reaction occurs in three phases (Figure 1). First, glucose attaches to a free amino acid (mainly lysine and arginine) of a protein, lipid or DNA, in a non–enzymatic way to form a Schiff base. A Schiff base is a compound that has a carbon to nitrogen double bond where the nitrogen is not connected to hydrogen. The initiation of this first step depends on glucose concentration and takes place within hours. If the concentration of glucose decreases, this reaction is reversible. During the second phase, the Schiff base undergoes chemical rearrangement over a period of days and form Amadori products (also known as early glycation products). The Amadori products are more stable compounds (hemoglobin A1c is the most well known), but the reaction is still reversible. If there is accumulation of Amadori products, they will undergo complicated chemical rearrangements (oxidations, reductions, and hydrations) and form crosslinked proteins. This process takes place in weeks or months and it is irreversible. The final brownish products are called AGEs and some of them have fluorescent properties. They are very stable, and accumulate inside and outside the cells and interfere with protein function [1,2]. Besides the Maillard reaction, other pathways can also form AGEs. For instance, the autoxidation of glucose and the peroxidation of lipids into dicarbonyls derivatives by an increase in oxidative stress is another pathway described for the formation of AGEs [3]. These dicarbonyl derivatives known as α-oxaldehydes (glyoxal, methylglyoxal (MG), and 3-deoxyglucosone) can interact with monoacids and form AGEs. The other well-studied mechanism for the formation of AGEs is the polyol pathway, where glucose is converted to sorbitol by the enzyme aldose reductase and then to fructose by the action of sorbitol dehydrogenase. Fructose metabolites (as fructose 3-phosphate) then are converted into α-oxaldehydes and interact with monoacids to form AGEs [4]. Thus, at least three pathways may form AGEs: The Maillard reaction; oxidation of glucose; and peroxidation of lipids and through the polyol pathway. Given these differing pathways, it is not surprising that AGEs are diverse in their chemical structure. Among the most widely studied AGEs are carboxymethyl-lysine (CML), pentosidine, and pyrraline, and, together with methylglyoxal (an α-oxaldehyde), they have been used as biomarkers for *in vivo* formation of AGEs [2,5,6]. CML (not fluorescent, not cross-linked AGEs) has been consistently used also as a biomarker for long-term protein damage and can be formed by the Maillard reaction and by α-oxaldehydes. As well as CML, pentosidine (a fluorescent protein crosslink) is formed by the Maillard reaction and by the α-dicarbonyl glyoxal, while pyrraline (not fluorescent, not cross-linked AGEs) is formed by the Maillard reaction [7]. 

**Figure 2:**
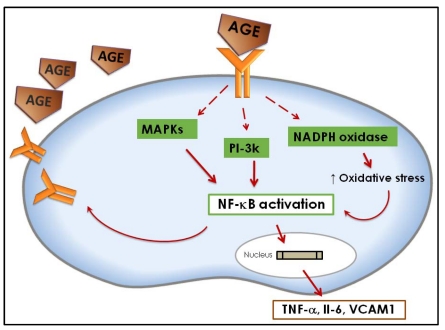
Mechanism of AGEs action at the cell level.

The deleterious effects of AGEs in different tissues are attributed to their chemical, pro-oxidant, and inflammatory actions [1,2]. The biological effects of AGEs are exerted by two different mechanisms: One independent of the receptor (damage of protein structure and extracellular matrix metabolism); or one involving the receptor for advanced glycation end products (RAGE) [2,8] (Figure 2). The interaction of AGEs with the receptor RAGE triggers the activation of the mitogen-activated protein kinases (MAPKs) and the phosphatidylinositol-3 kinase (PI3-K) pathways that will lead to the activation of the transcription factor NF-κB (nuclear factor kappa B). After activation, NF-κB translocates to the nucleus where it will activate the transcription of genes for cytokines, growth factors and adhesive molecules, such as tumor necrosis factor α (TNFα), interleukin 6 (Il-6), well known inflammation promoters, and vascular cell adhesion molecule 1 (VCAM1) [8,9,10,11,12]. NF-κB activation increases RAGE expression, creating a positive feedback cycle that enhances the production of inflammation promoters. In addition, AGE-RAGE interaction activates NAD(P)H oxidase (a complex of enzymes which produces superoxide) and when this complex is upregulated, it increases intracellular oxidative stress. The sudden increase in oxidative stress by NAD(P)H oxidase in response to AGE-RAGE interaction will also activate NF-κB [13,14,15]. 

## 3. Implications for Health

Accumulation of AGEs has been found in healthy aging persons, and this accumulation is higher during high glucose concentrations. Microvascular and macrovascular damage, seen in diabetes, is attributed to the accumulation of AGEs in tissues, but it is also associated with atherosclerosis, Alzheimer’s disease, end stage renal disease, rheumatoid arthritis, sarcopenia, cataracts, and other degenerative ophthalmic diseases, Parkinson’s disease, vascular dementia and several other chronic diseases [16,17,18,19]. For instance, Bar *et al.* have demonstrated differential increases of AGEs products in Alzheimer’s dementia and vascular dementia compared to controls [20]. It has also been suggested that AGEs are involved in the loss of bone density and muscular mass associated with aging [21]. We discuss briefly some of the health implications described in the older population.

### 3.1. Cardiovascular Diseases

The *in vivo* accumulation of AGEs over time contributes to changes in the structure and function of the cardiovascular system and presents as arterial stiffening, myocardial relaxation abnormalities, atherosclerotic plaque formation and endothelial dysfunction. Several authors have described some of the mechanisms for these changes. One of the proposed mechanisms involves additional cross-linking on collagen (whose normal structure already contains crosslinking) by glycation of its free amino acids. The collagen-AGEs cross-linking will produce stiffness of blood vessels. Sims *et al.* completed a histological study on 27 samples of post-mortem aortas from people with diabetes and controls and found a correlation between AGEs accumulation and aortic stiffness [22]. Another mechanism by which AGEs exert damage to the cardiovascular system is reduction of low-density lipoproteins (LDL) uptake by cell receptors. This occurs through glycation of the LDL particle on the apolipoprotein B and in the phospholipid components of LDL. The glycated LDL is more susceptible to cross-linking with collagen on the arterial wall than non-glycated LDL, and it is not taken up into the cell and accumulates. Macrophages uptake of these modified LDL lead to foam cell formation, and the development of atheroma [23,24]. Furthermore, decreasing in nitric oxide (NO) activity is another mechanism described by AGEs damaging the cardiovascular system. NO (a vasodilator) biosynthesis in the endothelium counteracts some of the mechanisms for atherosclerosis. Some authors proposed that AGEs reduce NO synthase (eNOS) half-life in the endothelium. For instance, Xu *et al.* found a decreased in eNOS activity after exposure to CML. They also found that after 30 minutes of exposure with CML-albumin, there was a reversible inhibition of endothelium and vascular response dependent on NO *in vivo* and *in vitro* [25,26,27,28]. 

Additional work supports the role of increased androgens during and after menopause as a risk factor for cardiovascular events in women, with an associated increase in AGEs. A study of 106 postmenopausal women found significant correlations between testosterone and free androgen indices *versus* AGEs after adjustment for age, body mass index, insulin resistance indices, and fasting glucose and insulin levels [29].

Therefore, the accumulation of AGEs could be explained by some of the cardiovascular changes associated with aging, such as vascular stiffening, diastolic dysfunction and endothelial dysfunction [24]. A study with long-term (24–30 weeks) administration of aminoguanidine (an inhibitor of AGE formation) showed prevention of the age-related cardiac hypertrophy and arterial stiffness [30]. It has also been found that CML, a predominant AGE, can serve as a predictor of cardiovascular mortality. Semba *et al.* studied a group of 559 women aged 65 and older for 4.5 years from the Women’s Health and Aging Study I (WHAS I). During this time 22% of the population died; 43.9% from cardiovascular disease. They measured CML as a marker for AGEs and found that the highest risk for dying of cardiovascular disease were for women in the highest quartiles of CML [31]. From these reports, it appears that high concentrations of AGEs could be a risk factor for cardiovascular disease, but further evidence is needed to support this statement. 

### 3.2. Sarcopenia

Loss of muscle mass and strength (sarcopenia) is a serious problem among older populations [32]. In accordance with recent studies, one third of women and half of men older than 60, have sarcopenia in the U.S. [33]. The pathogenesis of sarcopenia is multifactorial, and may include hormonal changes, increased oxidative stress and inflammation, changes in vasculature, and inactivity [34,35,36]. AGEs may also contribute to this condition by increasing oxidative stress and inflammation. Some studies have found a relation between AGEs and muscular function in older population. Haus *et al.* found that pentosidine concentrations were 200% higher in a group of older individuals with a mean age of 78 y (*n* = 22) compared with their younger counterparts with a mean age of 25 y (*n* = 20). The authors concluded that AGEs may contribute to the decline of muscular function observed in aging [37]. Dalal *et al.* conducted a study of older women to measure the AGEs concentration in blood and handgrip strength. Serum carboxymethyl-lysine (CML), the circulating soluble form of receptor for advanced glycation end products (sRAGE), endogenous secretory receptor for advanced glycation end product (esRAGE) and grip strength, were measured in 559 moderately-to-severely disabled women, age 65 and older, in the Women’s Health and Aging Study I in Baltimore. The authors found that women with higher concentrations of CML had less grip strength than women with lower CML concentrations. The authors concluded that women with higher AGEs have more muscle weakness [38]. These studies presented interesting results, but they are not conclusive of a causal relationship between AGEs and sarcopenia, and more studies will need to further address this health problem among older population. 

### 3.3. Renal Disease

The relationship between renal disease and AGEs has largely been studied in patients with type 2 diabetes mellitus, and to a lesser extent in older populations. Semba *et al.* [39] demonstrated that in an older population (*n* = 1008), elevated circulating AGEs were an independent predictor of renal function. The study was carried out in men and women, age 64 and older, participating in the InCHIANTI study in Tuscany, Italy. The results of the study demonstrated an elevated plasma concentration of CML independently associated with chronic kidney disease and the estimated glomerular filtration rate (an index of kidney function) at baseline, after three and six years of follow-up. These findings suggest that the potential adverse effects of AGEs on the kidney are applicable to the general population of older community-dwelling adults [39]. In another study of 548 women from the Women’s Health and Aging Study I in Baltimore, 51.6% of women had decreased glomerular filtration rate, which was associated with increased serum levels of CML and sRAGE. However, more follow-up studies on the elderly population are needed to establish if high levels of CML could predict decreased in renal function [40].

### 3.4. Alzheimer’s Disease

Although a definitive etiology for Alzheimer’s disease is unknown, oxidative stress has been identified as a primary risk factor for the disease. Both aging itself and the presence of AGEs are thought to be risk factors through their role in chemical, pro-oxidant, and inflammatory actions as previously described. A comparison of normal control and Alzheimer’s disease patients’ brain tissue found higher AGEs and RAGE expressions in age-matched controls [41]. In addition, there is evidence that RAGE mediates the blood-brain barrier transport of amyloid peptides in certain situations [42]. A recent review has described possible links between Alzheimer’s disease and diabetes, which include AGEs, advancing age, as well as oxidative stress and hypercholesterolemia, although exact mechanisms and relationships require additional research [43]. 

### 3.5. Diabetes

Hemoglobin A1c is the most widely recognized early glycation product, and is also used as an indicator of blood glucose management in those with diabetes. Hyperglycemia increases the glycation process, and is especially apparent in insulin independent tissues such as red blood cells, peripheral nerve tissue cells, endothelial cells, eye lens cells, and kidney cells [44]. It is also hypothesized that glycation of proteolytic enzymes in diabetes reduces their efficiency, resulting in more build up of glycated end products [44]. Not surprisingly, AGEs have also been implicated in delayed wound healing associate with diabetes, presumably through vascular, neurological, or intermediary metabolic modifications [45].

## 4. Exogenous Sources of AGEs

In addition to *in vivo* production, AGEs can also be found in cigarettes and in foods. The curing of tobacco leaves has been proposed as the source for compounds that can readily increase *in vivo* AGEs. Cerami *et al.* found that glycotoxins from cigarettes are inhaled into the alveoli, and then they are transported to blood stream or to lung cells where they can interact with other glycation products and contribute with AGEs formation [46]. 

### 4.1. Dietary AGEs

Heat has been used for treatment of foods to improve their safety, bioavailability and taste. In addition to these positive effects, overheating of foods can also provoke protein degradation and other deteriorative reactions [47]. Heat treatment in some foods results in promotion of the Maillard reaction, which adds desirable flavor, color and aroma. In the food industry, the Maillard reaction has been used for caramel production, coffee roasting, and bread baking among others. Some products of the Maillard reaction can be added to industrialized products such as sodas and juices among others [48]. There is growing evidence that the average Western diet is a plentiful source of exogenous AGEs. The AGEs content of a diet depends on the nutrient composition (foods rich in protein and fat have the highest content) and on the way food is processed [49,50]. AGEs formation can be rapidly accelerated by increasing the time and degree of exposure to heat and can be introduced into the body in heat–processed foods [47,49,50]. These findings were demonstrated using an AGE-specific, enzyme–linked immunosorbent assay (ELISA), and it was estimated that ≈10% of ingested immunoreactive AGEs are transported into circulation, two-thirds of which remain in the body, and are incorporated covalently in tissues. Only one third is excreted via the kidneys [51].

However, it has been controversial whether dietary AGEs are harmful to human health. One of the reasons for this controversy is that, as well as those found *in vivo*, Maillard reaction products formed in foods are heterogeneous and only a few have been characterized. Some of the products formed during this intricate reaction are furfurals, pyrralines and dicarbonyl compounds such as methylglyoxal. The products formed in the last reaction of this process are known as melanoidins in food science [52]. As mentioned before, regardless of the diversity of AGEs, CML has been reported as one of the most abundant *in vivo* and it was one of the first to be characterized in foods (milk and milk products). For this reason in most studies CML is chosen as a marker of AGEs in foods and *in vivo* [53].

Studies on the effects of AGEs from foods not only are limited to CML, but also to the melanoidins found in bread crust, bakery products and coffee. Some positive and negative effects of melanoidins have been studied. Ames *et al.* found that melanoidins increased the number of anaerobes, clostridia, and bifidobacteria in a culture of human fecal bacteria [54]. These results indicate that a mixture of melanoidins can stimulate growth of health-beneficial bacteria in the gut. Borrelli *et al*. found similar results showing that melanoidins from bread crust can promote growth of some bifidobacterias strains, indicating a possible potential prebiotic effect of bread crust melanoidins. Somoza *et al.* carried out a study in rats fed with malt and bread crust to measure the activity of chemopreventive enzymes such as glutathione-S-transferase (GST) and UDP glucuronyl-transferase (UDP-GT) [55]. The activity of GST in kidney increased by 18% on the group fed with bread crust, while UDP-GT in liver increased by 27%. The authors concluded that diet malt and dietary bread crust increased chemopreventive enzymes in rats. 

On the other hand, several studies, mostly with CML and MG, have shown that the intake of dietary AGEs modifies circulating AGEs levels in human subjects and animals with or without diabetes or renal disease. In a study with 90 healthy subjects, Uribarri *et al.* estimated the amount of AGEs from three days food records using a database with the AGEs content of certain foods. They found a significant correlation between the ingested AGEs and the plasma levels. A subgroup was exposed to a dietary restriction of AGEs and their plasma levels decreased as well. These results are similar to previous reports on patients with diabetes and renal failure patients [56]. Those findings support the view that the intake of dietary AGEs is an important contributor to the body AGEs pool [56,57,58,59,60]. Besides the endogenously formed AGEs, dietary AGEs have also been shown to act as RAGE ligands and activate major signal transduction pathways *in vitro* [11,61,62]. Dietary AGEs, together with those made endogenously, could promote a systemic glycoxidant burden, oxidant stress and cell activation, which increases vulnerability of target tissues to injury [63,64].

### 4.2. Dietary AGEs Metabolism

Several studies have focused on understanding the absorption, metabolism and excretion of dietary AGEs. Forster *et al.* carried out a study to try and understand the bioavailability and the kinetics of elimination of some Maillard products found on custard, pretzels and brewed coffee. They found that pretzel sticks are a rich source of pentosidine and pyrraline. The study was carried out with 18 healthy subjects who received specific amounts of these foods on a single day. Urinary excretion of pyrraline and pentosidine was measured by chromatographic methods for the following three days. The urinary excretion of both Maillard products increased after ingestion and the rate of recovery in urine was around 50% for pyrraline and around 60% for pentosidine. However, the metabolic fate of the pyrraline and pentosidine is unknown [65].

The mechanisms of intestinal absorption of AGEs are not yet well understood. A recent study trying to answer this question found that pyrraline is absorbed by the peptide transporter hPEPT1. This study is the first one addressing this question and studies on the absorption mechanism for more AGEs are needed [66].

A few studies about intestinal absorption of different AGEs have been conducted. However, the complete extent of absorption of each individual AGE is not well known. As mentioned before, the most studied dietary AGEs are CML, pyrraline and pentosidine. Several studies have shown different rates of absorption of each of them. However, their metabolic pathways have not been elucidated. More studies on this area are needed to understand the impact of dietary AGEs on health and aging.

## 5. AGEs in the Elderly

The serum levels of AGEs are dependant of endogenous production, exogenous intake and renal and enzymatic clearance, which together produce transient increases and decreases in serum AGEs levels. Several enzymes (glyoxalase I, II and carbonyl reductase) and a receptor (AGER1) have been shown to be part of a detoxification and counterregulation system against the prooxidant effects of glycation [67,68]. In addition, renal excretion eliminates excess of AGEs production under physiological conditions. Some authors have proposed that with aging as well as in some pathological conditions there is imbalance in this steady-state. This imbalance can be due to an increased endogenous production, or an increased exogenous intake that, in combination with lower renal AGEs clearance, leads to the accumulation of AGEs observed in older population [68,69]. 

Uribarri *et al.* investigated whether AGEs intake correlated with glycotoxin levels, markers of inflammation and oxidative stress (OS) comparing older *versus* younger healthy adults. They studied 172 healthy volunteers in two groups (18–45 years) and (60–80 years). The CML and MG derivatives were higher in the older group. The concentration of AGEs in serum correlated with levels of inflammation markers and OS. Additionally, the level of dietary glycotoxins correlated independently with CML and MG derivatives, as well as hsCRP. The association found between sCML and the homeostasis model assessment (HOMA an instrument to measure insulin sensitivity) levels of normal persons could be linked to metabolic processes, which may precede insulin resistance, diabetes mellitus, or vascular dysfunction at any age [70]. Vlassara *et al.* found similar results in a study with 325 healthy participants and 66 participants with kidney disease (CKD). Serum CML and MG were higher in the group of older participants, and serum CML correlated negatively with eGFR and positively with age [67].

The accumulation of AGEs in tissues may contribute to increased OS, and as a final result, impair organ function [16]. Indeed, within the complex the association between OS and aging ovarian follicles, AGEs may have an important role as they accumulate over the lifespan [71]. More recent work suggests a mechanism for this in that detoxification of an AGEs precursor is significantly diminished in older mice, allowing AGEs to increase [72]. Additionally, slowly diminishing renal function with age [73] could affect the ability to excrete AGEs. Uribarri *et al.* suggest this as a possible explanation as to why they found high AGE levels in the older group, even though the intake of dietary AGEs by the older age group was reduced [70]. More evidence is needed, but these results suggest the important role of dietary and circulating AGEs in chronic degenerative diseases, which are more prevalent in the elderly.

## 6. Strategies for AGEs Reduction

Some of the strategies studied to lower the extra load of AGEs are reducing dietary AGEs, pharmacological treatment and, more recently, exercise. 

### 6.1. Dietary Restriction of AGEs

Findings in several intervention studies, both human subjects and animals, indicate that the high intake of dietary AGEs contributes to tissue damage that can be prevented by dietary AGEs restriction. These intervention studies reduced dietary AGEs by decreasing the heat during the preparation of foods [74,75,76,77,78,79]. Sebekova *et al.* found that long-term consumption of AGEs in rats leads to a dose–dependent increase in proteinuria that overtime could induce renal damage [80]. In addition, the high long term consumption of AGEs has also been associated to higher levels of fasting glucose, insulin and serum AGEs, as well as increased AGEs localization and RAGE staining in ovarian tissue of rats [81]. In studies of mice, reduced dietary AGEs have been found to attenuate insulin resistance, increase the prevention of diabetes and, in diabetic mice, reduce diabetic vascular and renal complications, and improve impaired wound healing [74,75,76]. 

Whereas in human studies, Uribarri *et al.* demonstrated that intake of dietary AGEs by people with type 1 and 2 diabetes promotes the formation of pro-inflammatory mediators, leading to tissue injury [82]. Patients with uremia, with and without diabetes, in whom the intake of AGEs was reduced, showed reduced levels of inflammatory molecules such as TNF-α and high sensitivity C-reactive protein (hsCRP) [79]. In another study in patients with type 2 diabetes mellitus, decreasing the intake of AGEs for six weeks contributed to decreased levels of circulating AGEs and inflammatory markers [60]. The effects of reducing dietary AGEs have also been studied in nondiabetic peritoneal dialysis patients, a group that has very high AGE levels, and the results showed significant reduction in the levels of AGEs and C-reactive protein [79].

### 6.2. Role of Restriction of Dietary AGEs in Lifespan

It has been demonstrated that caloric restriction increases lifespan in *C. elegans* and mice. Several centenarian populations have been studied, and they have one thing in common: a lower caloric intake [83,84]. It has been postulated that positive outcomes of caloric restriction (CR) in mice could be explained in part by a decrease in the intake of AGEs and concomitant decrease of OS [85].

To investigate whether a reduction in CR would decrease the AGE intake, and whether this decrease could explain the benefits of CR, investigators studied three groups of mice assigned to one of three diets (*n* = 22 per group): CR diet, regular diet; or CR diet high in AGEs [86]. A longer lifespan in CR mice *versus* Reg or CR-high mice (median and maximal survival 13.2% and 6%, respectively) was reported. Additionally, survival in CR-high mice was shorter than in Reg mice. There was a significant increase of OS in the CR-high group and accelerated aging-related cardiovascular and renal disease and a shorter lifespan. In these studies, the high levels of AGEs in the CR-high diet compete with the benefits of CR, but the mechanism remained uncertain [86]. 

### 6.3. Exercise and AGEs

Published reports of research linking exercise and AGEs are sparse. One of the first studies in this subject suggested a decrease in levels of AGEs in exercise-trained diabetic rats as compared with sedentary diabetic rats. A possible explanation for the decrease is that adaptation to systemic physical exercise in diabetic animals affects not only enzyme-regulated metabolism but also non-enzymatic processes involving protein glycation. This study was published in Russian, so access was only to the abstract. Another study explored whether exercise prevented the age-associated changes in non–enzymatic cross-linking of myocardial collagen, and thus may improve cardiac performance in an animal model. In this study long-term exercise training appeared to attenuate age-related deteriorations in cardiac contractility and myocardial stiffness and was related to decreases in myocardial pathologic collagen cross-linking (AGEs cross-linked collagen) in old rats (*n* = 7) when compared with controls (*n* = 7) [87]. Boor *et al.* carried out a study in Zucker rats where one group (*n* = 8) had a training program for five weeks and the other group (*n* = 8) did not perform any exercise. It was found that the group with the training protocol had lower levels of CML in plasma, renal cortex and in glomeruli, in comparison with the sedentary group [88]. 

Few studies have explored the effects of exercise on AGEs on the human model. One of them showed the effect of Tai Chi in a healthy Malaysian population older than 45 years. The subjects were randomized either to practice Tai Chi two times per week or to a control group. Measures were taken at 0, 6, and 12 months. The subjects in the Tai Chi group had a decrease in concentration of AGEs and malondialdehyde MDA (a lipoxidation marker) after 12 months of intervention [89]. In another study, researchers reported the acute effect of exercise in subjects with coronary artery disease, finding no difference between the levels of RAGE before and after the training session [90]. Recently, Yoshikawa recruited seventeen healthy women (30–60 y) who participated in a lifestyle modification protocol for three months to measure changes in AGEs. The protocol aimed to increase physical activity among the intervention group, which was measured by a pedometer. An education session was given at the beginning of the study and participants attended supervised sessions once per week. Levels of CML decreased in the treatment group compared to the control group (*n* = 12) and CML decrease was negatively correlated with the number of daily steps [91]. 

### 6.4. Pharmacological Interventions

Several pharmacological agents have been studied as blockers of AGEs crosslinking or as blockers of their actions using cellular, animal and human models: benfotiamine (a B1-like vitamin with higher bioavailability), metformin, aminoguanidine, aspirin, and inhibitors of the renin-angiotensin system for example. Saha *et al.* showed that Candersatan, an angiotensin receptor blocker, administered for 12 weeks reduced levels of CML in the urine of patients with diabetic kidney disease [92]. Aminoguanidine, a scavenger of α-dicarbonyl, showed in some studies promising results as an inhibitor of AGEs formation. However, a clinical trial carried out in diabetic patients was stopped due to safety concerns and lack of efficacy. Patients presented secondary effects that included gastrointestinal disturbance, abnormal tests for liver function, and flu-like symptoms [93]. Additionally, metformin has shown to decrease circulating AGEs in patients with diabetes, as well as decreasing the activity of NFκB [94]. In a study of 22 women with polycystic ovary disease (PCOS), metformin therapy for six months resulted in a reduction of serum AGEs [95]. In a similar intervention with 21 women with PCOS, Orlistat, a lipase inhibitor, reduced serum AGEs after a high AGEs meal as compared to 15 women without PCOS by decreasing AGEs absorption [96]. A six month study evaluating the effect of calorie restriction and Orlistat found serum AGEs reduction in both the PCOS and obese groups, independent of body mass index changes [97]. Some of these agents are in preclinical or clinical phase trials, and it could be a long time before any of these treatments emerge as effective and safe therapeutic agents to inhibit and counteract AGEs effects.

## 7. Limitations of Dietary AGEs Studies

Formation of AGEs in foods is a complex process involving several reactions and many end products. Only a few of them have been characterized and measured in foods. Some of these products have fluorescence properties. By taking advantage of this property, some studies have measured AGEs using chromatography methods while other studies have measured AGEs by immunohistochemical techniques [53,98]. There is as yet no agreement among the different groups studying dietary AGEs as to which is the best. Recently Uribarri *et al.* published a food database with approximately 500 food items with CML content using ELISA [49]. This database represents a great tool to asses CML content of the diet of larger populations. However, more extensive research with other methodology is needed to validate CML content of this database. 

Intervention studies on the implications of reducing dietary AGEs have been performed mainly in patients with renal disease or diabetes. A few studies exploring the short term effects (2–4 weeks) of reducing dietary AGEs in healthy subjects have been performed mainly in young population [99,100]. Long-term clinical studies with older individuals are needed to determine the health effects of dietary AGEs on this population, but the methodological design of long-term studies represents a great challenge. One of the difficulties is achieving diets with different dietary AGEs content, but with similar content in other nutrients, such as heat-sensitive vitamins. Pouillart *et al*. demonstrated that diets with different dietary AGEs content but similar thiamine, vitamin E and other heat-sensitive vitamins content, are challenging but possible [101]. Future intervention studies addressing the impact of dietary AGEs will need to include diets with varying AGEs content, adequate content of all nutrients, as well as be attractive and tasteful to achieve high compliance during these long-term interventions. 

## 8. Conclusions

Although the data are not conclusive, the convergence of data from diverse experimental studies suggests an important role of AGEs in healthy aging, as well as chronic disease morbidity. Certainly the data are supportive that endogenous AGEs are associated with declining organ functioning. It appears that dietary AGEs may also be related. There are no conclusive results about the damaging effect of Maillard products from foods, but it appears from several studies that Maillard products coming from foods rich in protein and fat are more damaging than Maillard products from bread crust, and roasted coffee. Foods rich in protein and fat seem to have a higher content of CML and Methylglyoxal; in contrast, bread crust has lower content of these two AGEs. However, characterization of AGEs in food and biomarkers of these AGEs require additional research before reaching a conclusion. 

Although many promising pharmacologic anti-AGE therapies exist, their efficacy and safety are still under study. Research in this area is in the earliest phase, and a long time could pass before the U.S. Food and Drug Administration could approve a drug targeting AGE formation or modification.

As of today, restriction of dietary intake of AGEs and exercise has been shown to safely reduce circulating AGEs, with further reduction in oxidative stress and inflammatory markers. More research is needed to support these findings and to incorporate these into recommendations for the elderly population.
